# Reduced NLRP3 Gene Expression Limits the IL-1*β* Cleavage via Inflammasome in Monocytes from Severely Injured Trauma Patients

**DOI:** 10.1155/2018/1752836

**Published:** 2018-05-09

**Authors:** Shinwan Kany, Johann-Philipp Horstmann, Ramona Sturm, Katharina Mörs, Borna Relja

**Affiliations:** ^1^Department of Trauma, Hand and Reconstructive Surgery, Goethe University Frankfurt, 60590 Frankfurt, Germany; ^2^Department of Psychiatry and Psychotherapy, University Medicine Mainz, 55131 Mainz, Germany

## Abstract

**Objective:**

Traumatic injury or severe surgery leads to a profound immune response with a diminished functionality of monocytes and subsequently their IL-1*β* release. IL-1*β* plays an important role in host immunity and protection against infections. Its biological activation via IL-1*β*-precursor processing requires the transcription of inflammasome components and their activation. Deregulated activity of NOD-like receptor inflammasomes (NLR) like NLRP3 that leads to the maturation of IL-1*β* has been described in various diseases. While the role of other inflammasomes has been studied in monocytes, nothing is known about NLRP3 inflammasome after a traumatic injury. Here, the role of the NLRP3 inflammasome in impaired monocyte functionality after a traumatic injury was analyzed.

**Measurements and Main Results:**

Ex vivo-*in vitro* stimulation of isolated CD14^+^ monocytes with lipopolysaccharide (LPS) showed a significantly higher IL-1*β* secretion in healthy volunteers (HV) compared to trauma patients (TP) after admission. Reduced IL-1*β* secretion was paralleled by significantly lowered gene expression of NLRP3 in monocytes from TP compared to those of HV. Transfection of monocytes with NLRP3-encoding plasmid recovered the functionality of monocytes from TP regarding the IL-1*β* secretion.

**Conclusions:**

This study demonstrates that CD14^+^ monocytes from TP are significantly diminished in their function and that the presence of NLRP3 components is necessary in recovering the ability of monocytes to produce active IL-1*β*. This recovery of the NLRP3 inflammasome in monocytes may imply a new target for treatment and therapy of immune suppression after severe injury.

## 1. Introduction

Each year millions of people sustain injuries with the need for medical treatment, and around five million people died from them in 2013 [1]. Regarding severe traumatic injuries, a biphasic pattern of mortality is evident, as those patients who survive the initial injury are still at risk of death caused by immunological alterations ending in, for example, multiple organ failure in the later clinical course [2, 3]. Despite extensive research to address the immune response after trauma and as observed in intensive care units, the pathophysiology of immunological alterations is not yet fully understood, and the mortality remains high [4]. Experimental data links dysfunction of circulating monocytes to poor outcomes in patients with severe tissue injuries caused by trauma as well [5–7]. The biological immune response to tissue damage implies cytokine secretion which is triggered by either pathogen-associated molecular patterns (PAMP) like lipopolysaccharide (LPS) or damage-associated molecular patterns (DAMP) such as high mobility group box B1 (HMGB1) protein. In trauma, however, functional depression of monocytes leads to a suppressed production of interleukin- (IL-) 1*β* [6, 8]. On the other hand, lack of IL-1*β* is associated with immune suppression in critical illness and enhanced susceptibility to lethal infections as shown in mice IL-1 knockout models [9].

IL-1*β* undergoes a thoroughly controlled process of transcription, translation, and activation. Major sources of IL-1*β* include blood monocytes, macrophages, and dendritic cells among others [10, 11]. IL-1*β* messenger ribonucleic acid (mRNA) transcription is induced upon activation of Toll-like receptors (TLRs) by, for example, LPS or endogenous cytokines [12]. Additional stimuli like adenosine triphosphate (ATP), viral RNA, or pore-forming toxins activate the intracellular NOD-like receptor sensor molecule containing pyrin domain (NLRP) inflammasome and thereby the IL-1*β* converting enzyme caspase-1 to cleave IL-1*β*-precursor into its active form [13, 14]. As multiprotein complexes, inflammasomes consist of the effector protein caspase-1, an apoptosis-associated speck-like protein containing a caspase activation and recruitment domain (CARD) (ASC) and a sensor protein from the NOD-like receptor (NLR family) like NLRP1, NLRP3, or absent-in-melanoma (AIM)2 among others [15]. The presence of each inflammasome component is imperative for its formation and activation [15]. Unlike other inflammasomes, NLRP3 is triggered by a broader range of stimuli like uric acid crystals or asbestos and may, therefore, be more fundamental for the host defense [16, 17].

Impaired functionality of monocytes occurs early after trauma upon the admission of patients to the emergency departments as well as during the later clinical course [18, 19]. In monocytes isolated from patients with septic shock, the lack of NLRP1 mRNA correlated with the impairment of monocytes [20]. Our group showed that functional depression of monocyte from trauma patients went along with reduced NLRP1 mRNA expression and moreover that restoring NLRP1 via transfection recovered the functional activity of monocytes [19]. In this study, we examined if the NLRP3 inflammasome in monocytes isolated from trauma patients plays a mechanistically important role in reduced IL-1*β* secretion as well. To our knowledge, this has not been done before and could lead to the identification of crucial therapeutical targets against infections and organ failure in patients after severe trauma.

## 2. Patients and Methods

### 2.1. Ethics

This study was performed in the University Hospital Frankfurt of Goethe University. The study was approved by the institutional ethics committee and was performed in accordance with the Declaration of Helsinki and following STROBE guidelines [21]. In accordance with ethical standards, all enrolled subjects, healthy volunteers as well as patients, signed the written informed consent form by themselves or the written informed consent was obtained from the legally authorized representative.

### 2.2. Patients

Patients between 18 and 80 years of age with an Injury Severity Score (ISS) ≥ 16 and a history of acute blunt or penetrating trauma were included. Based on the Abbreviated Injury Scale (AIS) of 2008, the ISS was calculated [22, 23]. Exclusion criteria consisted of preexisting immunological disorders, immune-suppressive and anticoagulant medication, acute myocardial infarction, thromboembolic events, and/or lethal injury. Vital signs were measured upon arrival at the emergency department (ED), and blood samples were also obtained on admission to ED. Twenty patients with traumatic injury (trauma patients (TP)) and 10 healthy volunteers (HV) for comparison were included.

### 2.3. Blood Sampling

The blood samples were acquired immediately after the patient's admission to ED using ethylenediaminetetraacetic acid (EDTA) monovettes (Sarstedt, Nürmbrecht, Germany) and kept at room temperature due to subsequent functional assays.

### 2.4. Isolation of CD14^+^ Monocytes

Fresh blood samples from TP and HV were used for CD14^+^ monocyte isolation. Briefly, monocytes were isolated using Ficoll's density gradient (*d* = 1.077 g/mL, Biochrom AG, Berlin, Germany) and centrifugation at 600*g* for 20 min at room temperature [19]. Subsequently, the mononuclear cell layer was removed and washed in MACS buffer (2 mM EDTA, 0.5% bovine serum albumin (BSA) in phosphate-buffered saline (PBS, w/o Mg^2+^ and Ca^2+^)). The washing procedure was repeated twice. Then, by applying positive selection, anti-CD14-coated magnetic beads were used according to manufacturer's instructions (Miltenyi Biotec, Bergisch Gladbach, Germany). Flow cytometric analysis verified the purity of the isolated CD14^+^ cells (>97%). After isolation and purity determination, 1 × 10^5^ CD14^+^ monocytes/200 *μ*L RPMI-1640 (Seromed, Berlin, Germany; 48-well multidish plates, BD Bioscience, Franklin Lakes, NJ, USA) supplemented with 10% heat-inactivated fetal calf serum (FCS), 100 IU/mL penicillin and 100 *μ*g/mL streptomycin (Gibco, Karlsruhe, Germany) and 20 mM HEPES buffer (Sigma, Deisenhofen, Germany) were cultured for 1 h at 37°C under 5% CO_2_. Then, nonadherent cells were removed, and adherent CD14^+^ monocytes were washed again and cultured in 200 *μ*L RPMI-1640 with supplements as described above and before [19].

### 2.5. Ex Vivo-*In Vitro* Stimulation with LPS and IL-1*β* Release

CD14^+^ isolated monocytes were stimulated with LPS (10 *μ*g/mL, *E. coli* 0127: B8, Sigma) in 200 *μ*L RPMI-1640 with supplements per well and cultured for 24 h at 37°C and 5% CO_2_ as described previously [19]. The supernatant was removed and stored at −80°C after 24 hours. The samples were used for an IL-1*β* ELISA assay (Quantikine®, Human IL-1*β*/IL-1F2 Immunoassay ELISA, R&D Systems) according to the manufacturer's instructions. The adherent cells were used for RNA isolation and gene expression analyses.

### 2.6. RNA Isolation and Semiquantitative Reverse Transcription Polymerase Chain Reaction (qRT-PCR)

The RNA from cultured CD14^+^ monocytes was isolated using the RNeasy-system (Qiagen, Hilden, Germany) according to the manufacturer's instructions and as described before [19]. RNase-Free DNase Set was applied to remove any residual amounts of DNA according to the manufacturer's instructions (Qiagen), and both RNA quality and quantity were determined using the NanoDrop ND-1000 device (NanoDrop Technologies, Wilmington, DE, USA). RNA was stored at −80°C until cDNA synthesis and qRT-PCR as described previously [24]. Briefly, 100 ng of total RNA was reversely transcribed using the Affinity Script QPCR-cDNA Synthesis Kit (Stratagene, La Jolla, CA, USA) following the manufacturer's instructions. Gene-specific primers for human NLRP3 (NM_183395, UniGene number: Hs.159483, Cat. number: PPH13170A) and GAPDH (NM 002046, UniGene number: Hs.592355, Cat. number: PPH00150E) as reference gene were purchased from SABiosciences (SuperArray, Frederick, MD, USA). The PCR reaction was set up with 1x RT [2] SYBR Green/Rox qPCR Master Mix (SABiosciences) in a 25 *μ*L volume and a two-step amplification protocol with initial denaturation at 95°C for 10 min followed by 40 cycles with 15 s denaturation at 95°C, and 60 s annealing/extension at 60°C was applied. The specificity of amplification products was controlled by a melting-curve analysis. And qRT-PCR was carried out on a Stratagene MX3005p QPCR System (Stratagene).

Relative expression of target mRNA in each sample was calculated using the comparative threshold cycle (CT) method (ΔΔCT method) [25]. Here, the amount of target mRNA was normalized to the amount of housekeeping GAPDH to provide ΔCT and subsequently to a calibrator consisting of samples obtained from unstimulated control cells (fold change to unstimulated).

### 2.7. Reconstitution of NLRP3 in Isolated CD14^+^ Monocytes

Isolated CD14^+^ monocytes were transfected with 0.6 *μ*g/mL of either human NLRP3 (HS_NLRP3_IM_1, gene ID: 114548, NM_001079821, EIM0044989) or the positive/plasmid control (PC, *n* = 6) CDC2 (delivered with the kit) expression plasmids (vector backbone pQE-TriSystem-6, QIAgenes Expression Kit Insect/Mammalia, Qiagen, Hilden, Germany) in the FCS-free culture medium. And the transfection reagent Attractene (5:1 DNA, Qiagen) was used. The medium was substituted with RPMI-1640 culture medium supplemented as described above two hours later. 48 hours later, cells were challenged with LPS (10 *μ*g/mL, *E. coli* 0127: B8, Sigma) and incubated for additional 24 hours before cell extracts for gene and protein as well as supernatants for IL-1*β* analyses were obtained. The viability of isolated cells gradually decreased over time; however, staining with propidium iodide (PI, Sigma Aldrich Ltd.) and subsequent microscopical evaluation have shown that there was a minimal cell death upon transfection. Monocytes that regularly displayed at least 75% viability were applied for the experiments.

### 2.8. Verification of the Transfection via Immunohistological Staining

Isolated monocytes from healthy volunteers were used to monitor the transfection efficiency. In short, 48 hours after transfection, isolated cells were washed (PBS, w/o Mg^2+^ and Ca^2+^) and fixed using 100 *μ*L solution A (FIX & PERM® Cell Fixation & Permeabilization Kit, ADG Bioresearch GmbH, Kaumberg, Austria) for 10 min. Then, cells were washed again and incubated with a mouse monoclonal Penta-His antibody directed against His-tagged recombinant protein (Qiagen) and resuspended in 100 *μ*L solution B (from the same kit) for 15 min at room temperature. After another washing procedure with 0.5% BSA in PBS, cells were stained with a secondary NL557 antimouse antibody (R&D Systems, Minneapolis, USA) for 30 min. After a final PBS washing step, cells were microscopically analyzed using the Axio Observer Z1 (Zeiss, Göttingen, Germany).

### 2.9. Protein Isolation and Western Blotting for NLRP3 Components

After the transfection process, the cells were lysed in the lysing buffer (50 mM Tris base-hydrochloric acid (Tris-HCl), pH 7.4; 150 mM sodium chloride (NaCl); 1% tergitol-type NP-40; and 0.25% Na-deoxycholate) containing protease and phosphatase inhibitors (1 mM phenylmethylsulfonyl fluoride (PMSF); 1 mM ethylenediaminetetraacetic acid (EDTA); aprotinin, pepstatin, and leupeptin (1 *μ*g/mL each); 1 mM sodium orthovanadate (Na_3_VO_4_); and 1 mM sodium fluoride (NaF)) at 4°C. Then, a centrifugation for 30 min at 4°C at 20.000 ×g followed. Supernatants were stored at −80°C for subsequent western blotting analysis.

Electrophoresis on 12% polyacrylamide SDS gels was used to separate lysates (50 *μ*g protein) which were then transferred to Hybond-C Extra supported nitrocellulose membranes (Amersham-Buchler, Braunschweig, Germany). NLRP3 was detected using mouse monoclonal NLRP3/NALP3 (human), mAb (Nalpy3-a) antibody (Enzo Life Sciences, NY, USA), and His by using mouse monoclonal His-probe antibody (H-3) (all Santa Cruz Biotechnology, Santa Cruz, CA, USA). *β*-actin as internal loading control was detected by a mouse anti-*β*-actin antibody (Sigma, Taufkirchen, Germany). Blots were blocked (10% nonfat dry milk in 1 mM Tris, 150 mM NaCl, pH 7.4) for 1 hour, then incubated for 1 hour at RT with primary antibody (diluted according to manufacturer's instructions in blocking buffer with 0.5% Tween 20 and 0.5% BSA), and then incubated for another hour with horseradish peroxidase-conjugated secondary antibodies (Santa Cruz Biotechnology) diluted 1 : 1000 in blocking buffer with 0.5% Tween 20 and 0.5% bovine serum albumin at RT as described before [19]. ECL™ western blot detection reagents (GE Healthcare, Munich, Germany) were used for protein detection. The signals were digitized using the FUSION-FX7 machine and Bio-1D software (Peqlab Biotechnologies, Erlangen, Germany).

### 2.10. Statistical Analysis

Kolmogorov-Smirnov test was used to verify the normality of data. Student's *t*-test with Welch correction and one-way analysis of variance (ANOVA) with Dunn post hoc test were applied for comparison among different groups. A *p* value below 0.05 was considered statistically significant. Data are given as mean ± standard error of the mean (SEM). And statistical analysis was performed with GraphPad Prism 6.0 software (GraphPad Software Inc. San Diego, CA).

## 3. Results

### 3.1. Study Subjects Represent Severely Injured Trauma Patients

In this study, 20 patients with traumatic injury as well as 10 healthy volunteers were enrolled. Most of the patients were male (15 TP, 75% versus 7 HV, 70%, Table 1). The mean age of study subjects was 38.95 ± 3.50 years for TP and marginally younger in HV with 35.80 ± 3.99 years (Table 1). The enrolled cohort of patients had severe injuries as the mean ISS was 30.5 ± 1.80 points. The stay at the intensive care unit (ICU) was 12.90 ± 1.50 days. The mean hospital duration was 20.95 ± 2.99 days (Table 1). This cohort of severely injured trauma patients is comparable to preceding studies and represents this cohort of patients with severe and major injury as often observed in the ICU [26–28].

### 3.2. Lowered LPS Response in Isolated CD14^+^ Monocytes Is Paralleled by Decreased NLRP3 Gene Expression after Trauma

**Figure 1:**
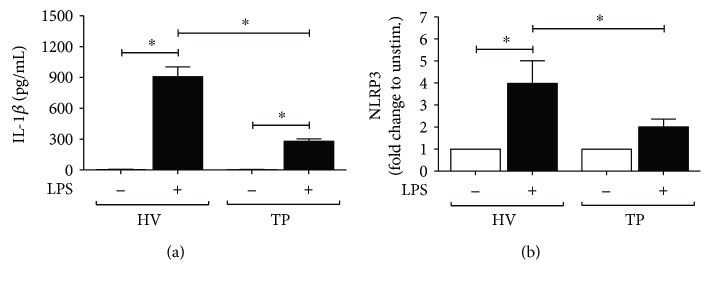
Trauma reduces IL-1*β* secretion and NLRP3 expression in isolated CD14^+^ monocytes. (a) Isolated CD14^+^ cells from peripheral mononuclear blood obtained either from trauma patients (TP, *n* = 20) on admission or healthy volunteers (HV, *n* = 10) were incubated and treated as described in Patients and Methods. IL-1*β* secretion (pg/mL) after stimulation with lipopolysaccharide (LPS) was determined by ELISA. (b) Gene expression analysis of NLRP3 as fold change to unstimulated CD14^+^ monocytes which were isolated and treated with LPS as described. Data are shown as mean ± SEM. ^∗^*p* < 0.05* versus* indicated.

To determine that isolated monocytes react as described before in own studies and in literature, the IL-1*β* secretion capabilities after LPS challenge were measured using an ELISA assay [19, 20]. The ex vivo-*in vitro* LPS-stimulated IL-1*β* secretion was significantly enhanced in HV and TP after LPS challenge compared to the corresponding nonstimulated cells (*p* < 0.05, Figure 1(a)). Compared to the IL-1*β* response of HV isolated, CD14^+^ monocytes from TP released significantly less IL-1*β* upon LPS stimulation (*p* < 0.05, Figure 1(a)). Therefore, the isolated CD14^+^ monocytes of our cohort display the previously described impaired function of these cells after trauma.

To verify that the diminished function of CD14^+^ monocytes after LPS challenge in TP is associated with NLRP3, NLRP3 gene expression was measured. Gene expression of NLRP3 in monocytes from HV was significantly increased compared to that in unstimulated cells (*p* < 0.05, Figure 1(b)). In isolated CD14^+^ monocytes from TP, LPS stimulation did not lead to a significantly increased NLRP3 gene expression, although there was a trend to an increase compared to nonstimulated controls (Figure 1(b)). When comparing both LPS-challenged monocytes from HV and TP, NLRP3 gene expression was significantly decreased in CD14^+^ monocytes from TP versus HV (*p* < 0.05, Figure 1(b)). This data underlines diminished immune regulatory capabilities of the investigated monocytes from patients with severe injury and directly indicates an association between reduced IL-1*β* response after LPS challenge and NLRP3 gene expression.

### 3.3. NLRP3 Transfection of Isolated CD14^+^ Monocytes

**Figure 2:**
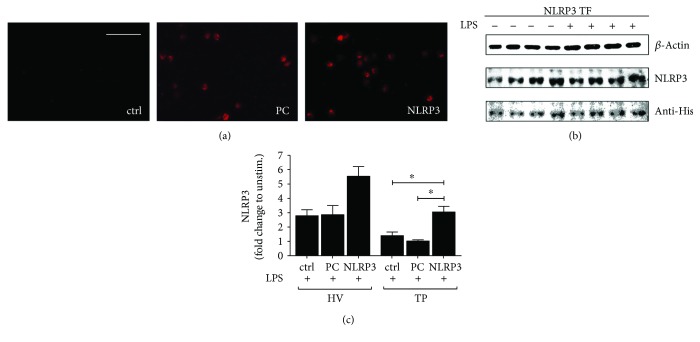
Transfection of isolated CD14^+^ cells with the NLRP3 plasmid increases NLRP3 gene and protein expression. (a) Immunohistological staining of human CD14^+^ monocytes isolated from healthy volunteers (HV). Left panel presents nontransfected cells (ctrl), middle panel displays CD14^+^ cells transfected with plasmid control (PC), while the right panel shows cells transfected with the NLRP3 plasmid. Representative photos are shown. (b) Isolated CD14^+^ monocytes were transfected with NLRP3 plasmid and either untreated or treated with lipopolysaccharide (LPS). Protein extracts were isolated from the cells and analyzed via western blotting as described in Patients and Methods to detect indicated proteins. Representative blots of four independent experiments are shown. (c) Isolated CD14^+^ monocytes from either HV or TP were transfected with NLRP3-encoding plasmid (NLRP3) or plasmid control (PC) or remained untreated (ctrl). Cells were then stimulated with LPS and gene analysis of NLRP3 was represented. Data are shown as mean ± SEM. ^∗^*p* < 0.05* versus* indicated.

Isolated CD14^+^ cells were transfected with either plasmid coding for NLRP3 or plasmid control (PC) to understand the role of NLRP3 inflammasome elements for monocyte functionality. In Figure 2(a), positive red staining of the His-tag in isolated monocytes verifies the expression of encoded genes in plasmid by recognizing the His-tag fused to control vector. The left panel of immunostaining displays no positive tag staining in nontransfected cells, while the middle panel shows positive staining of PC and indicates, therefore, an expression of the PC plasmid (Figure 2(a)). In the right panel, the NLRP3 expression is confirmed via positive red staining (Figure 2(a)).

Protein expression of NLRP3 was verified by western blotting of whole-cell protein extracts of isolated CD14^+^ monocytes after NLRP3 transfection and LPS stimulation or of ctrl (Figure 2(b)). Due to the limited availability of biomaterial from TP at admission to ED, this assay was performed only for monocytes of HV. A His-tag antibody was utilized to identify positive bands with a similar size as of those that were detected with NLRP3 antibody after transfection. Successful transfection of CD14^+^ cells from HV with NLRP3 is confirmed by this result (Figure 2(b)).

To verify the transfection efficiency, NLRP3 gene expression was determined after transfection in CD14^+^ cells from TP as well as HV (Figure 2(c)). Transfection with NLRP3 led to a significant rise of NLRP3 gene expression in TP and HV when compared to nontransfected or PC-transfected controls after LPS stimulation (*p* < 0.05, Figure 2(c)). Transfection with the PC was not significantly distinctive in its NLRP3 gene expression compared to control monocytes after LPS stimulation (Figure 2(c)). Transfection with NLRP3 in isolated monocytes from TP at ED admission did not reach gene expression levels of NLRP3 as compared to HV upon NLRP3 transfection, but the difference was not significant between those two groups (Figure 2(c)).

### 3.4. IL-1*β* Release after LPS Stimulation Is Recovered after NLRP3 Transfection of CD14^+^ Monocytes

**Figure 3:**
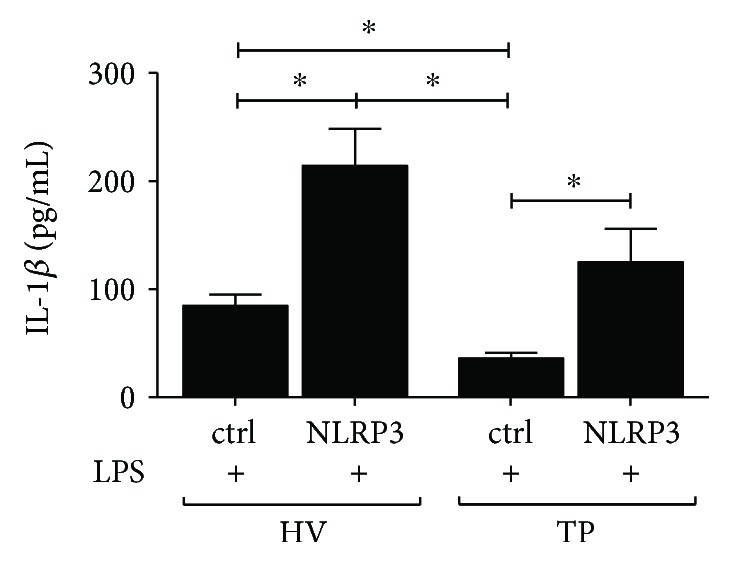
Transfecting CD14^+^ monocytes with NLRP3 recovers their functionality. CD14^+^ monocytes were isolated either from healthy volunteers (HV) or from trauma patients (TP) after the admission. Subsequently, cells were transfected with the NLRP3 expression plasmid or with plasmid control (ctrl) before stimulation with lipopolysaccharide (LPS). In the supernatants, secretion of IL-1*β* was determined by ELISA. Data are shown as mean ± SEM. ^∗^*p* < 0.05* versus* indicated.

To investigate how isolated monocytes from TP transfected with NLRP3-encoding plasmid will perform in regard to functionality, CD14^+^ cells were treated with LPS after transfection, and IL-1*β* secretion was measured. This was also implemented for monocytes from HV as control. The functionality of nontransfected cells with regard to IL-1*β* release was significantly decreased in ctrl monocytes from TP compared to ctrl monocytes from HV (*p* < 0.05, Figure 3). In NLRP3-transfected cells from HV, the IL-1*β* secretion after LPS stimulation was significantly higher compared to nontransfected monocytes from HV or TP, respectively (*p* < 0.05, Figure 3). The transfection of isolated CD14^+^ cells from TP with NLRP3 gene-encoding plasmid significantly enhanced IL-1*β* release after LPS challenge compared to that of nontransfected cells from TP (*p* < 0.05, Figure 3). This data indicates that NLRP3 transfection of functionally depressed monocytes after trauma enhances their IL-1*β* secretory capability.

## 4. Discussion

Monocytes constitute an integral part of the innate immunity defense as they early detect PAMP or DAMP via their pattern recognition receptors (PRR). The intracellular family of NOD-like receptors (NLR) as PRR is essential in activating host response to pathogens [29]. A prominent member of the NLR family is the receptor NLRP3 which is part of the NLRP3 inflammasome, an intracellular multiprotein complex that cleaves inactive IL-1*β* precursor to its active form via caspase-1 [17]. The cytokine secretion by monocytes is also required for the activation of adaptive immunity through T cells [29]. Severe injury or surgery can lead to compromised host defense ensuing sepsis and multiple organ failure [30, 31]. Here, it became evident that impaired capability of monocytes to secrete cytokines predicted high mortality caused by (infectious) complication after severe trauma [8, 18, 32]. Therefore, the inflammasome complexes became a major focus of research in recent years. With regard to trauma, NLRP1 has been studied by several groups. For instance, Fahy et al. described that low levels of mRNA for NLRP1 and caspase-1 in monocyte ex vivo were linked to sepsis and survival in septic shock [20]. Our group described a diminished functionality of monocytes in severely injured trauma patients and uncovered in ex vivo-*in vitro* analyses that reduced NLRP1 gene expression was a contributing factor to monocyte suppression after trauma [19]. The present study reveals that the NLRP3 inflammasome is apparently affected by trauma as well and diminished in its function due to decreased NLRP3 gene expression after trauma (Figure 1(a)). Furthermore, downregulated NLRP3 gene expression was paralleled by a decreased IL-1*β* response after LPS challenge. As described by others before, NLRP3 inflammasome is essential in the synthesis of mature IL-1*β*, which can be used ex vivo to asses NLRP3 activity in human monocyte-derived cells [10]. However, there is data conflicting our findings. A Brazilian group reported increased ex vivo NLRP3 gene expression in patients with septic shock, while NLRP1 and NOD1 were decreased [33]. However, they used peripheral blood mononuclear cells that include lymphocytes and NK cells as well, a difference that may explain the divergence to our data [33].

In the 1960s, Muckle and Wells first described a disease with transient attacks of fever, urticaria, and deafness in a family in Derbyshire, England [34]. Hoffman later described a related disease called “Familial Autoinflammatory Cold Syndrome” that became symptomatic when patients were exposed to cold temperatures [35]. The underlying genetic mutation coded for a protein that was termed cryopyrin and later NLRP3 [35]. Since then, NLRP3 mutation and hyperactivation have been linked to gout, diabetes type 2, familial Mediterranean fever, and rheumatic disease among many others [36]. However, in terms of traumatic injury, the activation of NLRP3 is essential for tissue repair via the so-called “regenerative inflammation” [37]. Furthermore, NLRP3 knockout mice models display a compromised host defense against *Candida*, *Toxoplasma gondii*, or *Salmonella* among others [38–40]. Despite all this research, the modulation of NLRP3 gene expression in traumatic injury has been scarcely studied. Effective assembly of the NLRP3 inflammasome complex and subsequent release of IL-1*β* requires the components to be fully available intracellularly [15]. Whole-blood assays by Singh et al. revealed enhanced NLRP3 mRNA levels in patients with septic shock that correlated with severity of the illness [41]. Nonetheless, the contribution of other cells than monocytes to the NLRP3 mRNA levels has to be taken into account, and additionally, the study population was rather small (*n* = 29) [41]. Another interesting work by a German group investigated inflammasome modulation in human monocytes after cardiopulmonal resuscitation (CPR) [42]. They presented data of elevated NLRP3 mRNA levels in monocytes 24 hours after CPR, while NLRP1 levels were diminished [42]. This correlates with further above-described findings of Fahy et al. Yet, when comparing the 30-day survival, those patients that had lower levels of NLRP3 mRNA in monocytes 48 hours after CPR had significantly lower survival in the 30-day period [42]. This provides insight into a protective role of NLRP3 in critically ill patients. The mode of injury (cardiac arrest and CPR versus severe traumatic injury) may explain our findings of lower NLRP3 mRNA levels in TP after admission. It is also worth noting that Asmussen et al. measured mRNA levels only and did not study the cells further.

In this study, we show that a reconstitution of the lacking NLRP3 in human monocytes from trauma patients can recover the NLRP3 gene expression and, apparently, thereby provide the function to secrete active IL-1*β* (Figures 2(a)–2(c) and 3). This is a novel finding that to our knowledge has not been reported before. Similar findings have been shown by our group for NLRP1 inflammasome [19].

However, there are limitations that have to be considered. First, our study population was rather small (*n* = 20), and further protein analyses were not possible due to limited biomaterial of trauma patients. Secondly, transfection of CD14^+^ monocytes from healthy volunteers led to an increased NLRP3 activity after LPS challenge just as well as in monocytes from trauma patients. Recent research of the aforementioned NLRP3 mutation syndromes may provide an explanation. It has been shown that patients with NLRP3 mutation display a maximally increased IL-1*β* secretion after LPS challenge [43].

While our group investigated NLRP1 before and NLRP3 inflammasome in this study, further research in this field may provide new perspectives in the treatment of critically ill patients. Larger study population and subpopulation analyses of septic and septic shock patients may reveal a certain pathology that may benefit from enhanced inflammasome activity. It also remains to be evaluated in preclinical *in vivo* models if an ex vivo transfection of monocytes and their subsequent retransfusion will lead to improved outcomes. In summary, associative data from this study indicate that the contribution of NLRP3 inflammasome to host defense after trauma is apparently larger than previously anticipated.

## Figures and Tables

**Table 1:** Patient's characteristics. ctrl: healthy volunteer controls; d: days; ICU: intensive care unit; ISS: Injury Severity Score; n.s.: not significant; TP: trauma patients; vs.: versus; y: years.

Characteristics	TP (*n* = 20)	ctrl (*n* = 10)	*p* value (TP versus ctrl)
Age (y)	38.95 ± 3.50	35.80 ± 3.99	n.s.
Sex (male, *n*)	15	7	n.s.
ISS	30.5 ± 1.80	—	
ICU stay (d)	12.90 ± 1.50	—	
Hospital stay (d)	20.95 ± 2.99	—	

## Data Availability

The data used to support the findings of this study are available from the corresponding author upon request.

## Abbreviations

AIS: Abbreviated Injury Scale
ASC: Apoptosis-associated speck-like protein containing CARD
BSA: Bovine serum albumin
CARD: Caspase activation and recruitment domain
CASP1: Caspase-1
CD: Cluster of differentiation
ctrl: Control
DAMP: Damage-associated molecular pattern
ED: Emergency department
ELISA: Enzyme-linked immunosorbent assay
EDTA: Ethylenediaminetetraacetic acid
GAPDH: Glyceraldehyde 3-phosphate dehydrogenase
HV: Healthy volunteers
ICU: Intensive care unit
IL: Interleukin
ISS: Injury Severity Score
LRR: Leucine-rich repeats
LPS: Lipopolysaccharide
mRNA: Messenger ribonucleic acid
NLR: NOD-like receptor
NLRP: NLR sensor molecule containing pyrin domain
PBS: Phosphate-buffered saline
PAMP: Pathogen-associated molecular pattern
PC: Plasmid control
PRR: Pattern recognition receptor
PYCARD: Gene encoding for ASC
TP: Trauma patients.
